# Editorial: Microbial strategies for phytoremediation enhancement

**DOI:** 10.3389/fmicb.2026.1917284

**Published:** 2026-07-27

**Authors:** Prayad Pokethitiyook, Toemthip Poolpak, Kwang Mo Yang, Pankaj Kumar Arora

**Affiliations:** 1 Faculty of Science, Mahidol University, Ratchathewi, Thailand; 2 ASEAN Institute for Health Development, Mahidol University, Salaya, Thailand; 3 Department of Plant Science, Mahatma Jyotiba Phule Rohilkhand University, Bareilly, India

**Keywords:** AI, biodegradation, heavy metal, omics, phytoremediation

The rapid development of industrialization, urbanization, mining operations, and agricultural activities has led to the accumulation of hazardous contaminants in both terrestrial and aquatic ecosystems (Sharma et al., 2023). Heavy metals, hydrocarbons, pesticides, pharmaceuticals, dyes, and emerging pollutants have become a threat to the environment due to their toxicity, bioaccumulative properties, and persistent behavior (Karnwal et al., 2024). Though traditional remediation methods are effective in some situations, they are costly and energy-consuming and have negative impacts on the environment (Garg et al., 2025). Phytoremediation becomes an environmentally friendly solution to remediate contaminated sites (Sahoo et al., 2025). The effectiveness of phytoremediation, however, may be hindered by inefficient plant growth, low availability of contaminants, phytotoxicity, and unfavorable environmental factors. Recent research indicates that microorganisms have a crucial effect in overcoming such barriers (Pradhan et al., 2025).

Microbially assisted phytoremediation is an innovative and interdisciplinary approach that combines aspects of plant biology, microbiology, soil science, environmental biotechnology, and molecular ecology (Saha et al., 2021). Microbial communities residing in the rhizosphere, endosphere, and phyllosphere affect plant growth and biodegradation of pollutants by several direct and indirect means (Simmer and Schnoor, 2022). Plant growth promoting rhizobacteria, arbuscular mycorrhizae, endophytic fungi and bacteria, cyanobacteria, and microbial consortia play important roles in contaminant uptake, detoxification, sequestration, and biodegradation. Microbes assist in the availability of nutrients, regulation of phytohormones, reduction in oxidative stress, and increased tolerance of plants in polluted environments (Basit et al., 2021).

This Research topic on “*Microbial strategies for phytoremediation enhancement*” emphasizes recent developments in the area of microbe-enhanced phytoremediation and environmental biotechnology involving research ranging from field-based rhizospheric microbiome research to research based on multi-omics, machine learning, and genetic engineering methodologies. As a whole, these studies show how microbes and their capabilities can be used for enhancing the performance of plants under environmental stresses and facilitating the degradation process of contaminants.

Rhizospheric bacteria act as biochemical engines of phytoremediation. They not only mobilize toxic metals but also rapidly degrade complex organic matters. They also promote plant growth through nutrient mobilization, phytohormone production and stress alleviation. The synergetic interaction between plants and rhizospheric microbes plays a significant role in bioremediation of toxic metals and organic compounds. Doku et al. examined the contribution of rhizosphere microbiomes to the phytoremediation capacity of *Leucaena leucocephala* in gold mining tailings in Ghana. *Leucaena leucocephala* exhibited remarkable resistance to high concentrations of heavy metals (>10,000 mg/kg Fe and Mn) under acidic conditions (pH 4.57–5.97), whereby maximum metal content was achieved at the final harvest, with iron content being as high as 14,605 mg/kg in shoots and manganese content of 12,279 mg/kg in roots. High metal content decreased overall bacterial diversity, yet bacteria resistant to metals such as Actinobacteria, Proteobacteria, and Acidobacteria comprised bacterial communities in all the soil samples. The genera of *Arthrobacter, Gaiella, Skermanella*, and *Chelatococcus* were positively correlated with metal accumulation while performing their ecological roles in the rhizosphere. *Nocardioides* and *Streptomyces* were dominant species in the early stages of colonization, indicating their nutrient and metal stress responses.

Further evidence of the benefits of plant growth-promoting rhizobacteria is provided by Sun et al. They highlight the effectiveness of a specific rhizobacterium in preventing toxic cadmium (Cd) accumulation in edible crops while simultaneously boosting plant growth. Researchers utilized the manganese-oxidizing rhizobacterium *Exiguobacterium acetylicum* 4-3-1 to protect spinach plants exposed to Cd stress (10.5 mg/kg). The bacterium successfully removed 73.74% of free CdCl2. In the spinach plants, it increased total biomass by 184.3% and chlorophyll content by 33.99%, while drastically reducing the toxic Cd concentration in the leaves by 53.07%. The bacterium protects the plant through a two-pronged approach: (i) intrinsic (Internal): it alters the plant's gene expression by boosting pathways related to photosynthesis and energy, while actively suppressing the genes responsible for transporting heavy metals into the plant. (ii) Extrinsic (External): it oxidizes manganese to form oxides that immobilize the Cd in the soil. It also improves the root microbiome by increasing the presence of beneficial bacteria like Bacillales.

The significance of endophytic microorganisms in heavy metal remediation is demonstrated by Yang et al. They analyzed the efficiency of three endophytic bacteria isolated from *Sorghum sudanense* for the enhancement of its ability to decontaminate soils with chromium contamination. Bacterial inoculations led to an increase in growth parameters of plants, such as height, root length, and shoot weight. Moreover, the inoculation had the effect not only on plant growth but also on reducing chromium accumulation and mitigating the negative impact of heavy metals on the organism, as was shown by the increased chlorophyll content and decreased content of malonaldehyde (MDA). Inoculation contributed to the control of activity of antioxidant enzymes and the improvement of soil properties, as the organic matter level was increased, and positive microbial communities were developed. Finally, the results obtained demonstrate great prospects of using endophytic bacteria for the enhancement of plant resistance and accelerated phytoremediation processes in heavy metal-contaminated soils.

The integration of artificial intelligence and systems biology into environmental remediation is addressed by Okafor et al. They investigated the issue of how machine learning and multi-omics integration can greatly enhance conventional bioremediation methods applied to hydrocarbon-polluted environments. The application of supervised machine learning algorithms (such as neural networks and support vector machines) together with multi-omics analysis provides an effective way of making predictions. The technology enhances the effectiveness of the purification process by predicting the most suitable plant species and microorganisms for particular environmental conditions. It also identifies the key genes, enzymes, and biochemical pathways involved in hydrocarbon degradation.

Expanding the concept of biological stress tolerance, de Figueras et al. investigated the possibility of increasing plant tolerance to UV-B radiation by introduction of four genes of extremophiles living in high altitude saline lakes (Argentina) and salt pans (Spain) into Arabidopsis thaliana. The chosen genes (coding for one TATA-box binding protein and three hypothetical proteins) were known to protect UV-sensitive E. coli (recA mutant) against UV rays. The transgenic plants were exposed to UV-B and UV-C (4.5 kJ·m^−2^) irradiation with the latter being used because of more damaging effect and cross-tolerance was checked by sodium perchlorate (3.67–7.34 g/L) which causes oxidative stress at higher concentration than in previous experiments. Main results: the transgenic plants showed increased tolerance to UV radiation and were able to grow in the medium with the higher content of perchlorate which suggests the contribution of these extremophile genes into the wide stress tolerance. Such an approach is a prospective way of increasing crop yield and stability due to increasing of UV radiation caused by ozone layer destruction and pollution.

These contributions to the Research Topic serve to help move our knowledge of the microbial involvement in phytoremediation forward. With the transition from broad observations to specific validations through metabolism and metagenomics, this body of research lays the foundation for future work by environmental scientists and engineers in more effective biologically based environmental remediation approaches. Thank you for your contributions on these fascinating biological collaborations.

The individual contributions to this Research Topic clearly represent an important step forward in microbe-assisted phytoremediation, since they collectively provide evidence that specific microbial communities, beneficial plant–microbe interactions coupled with the integration of novel technologies can enhance remediation capabilities of contaminated environments. The studies reported here strongly suggest that the acceleration of plant growth and tolerance to abiotic stress mediated by rhizosphere microbiomes, endophytic organisms, and plant-beneficial bacteria can also reduce metal translocation, facilitate immobilization and transformation of heavy metals and organic pollutants. In addition, the integration of multi-omics approaches, machine learning and genetic engineering enhance our insights into the molecular basis of plant–microbe interaction and can lay a foundation for rational design-based predictive phytoremediation strategies.

These contributions also indicate several leading challenges and research priorities as well. A key challenge is consistently translating laboratory and greenhouse outcomes to long-term field applications across diverse climatic and edaphic zones. Research in future work should focus on the stability, resilience and ecological safety of the introduced microbial inoculants or engineered plant-microbe systems in natural environmental conditions. Multi-omics data integration with AI-based approaches to define key microbial functions along with their predictive power over remediation outcomes and optimize microbial consortia for site-specific applications is another area that would need more attention from the research community. In addition, increased knowledge of microbial community assembly and interspecific interactions between microbes and the plant genotype-dependent recruitment of beneficial microbes will be especially important for developing resilient phytoremediation systems. Addressing regulatory, biosafety and scalability barriers will also be key to large-scale deployment of these technologies.

Altogether, this Research Topic illustrates that the future of phytoremediation is being redefined when microbial ecology is coupled to systems biology, synthetic biology and data-driven approaches. These studies relate the fundamental science and practical environmental applications and are a firm foundation for the development of sustainable, cost-effective, and climate-resilient bioremediation technologies that can tackle increasingly severe environmental pollution issues.
